# Serum concentration of appetite‐regulating hormones of mother–infant dyad according to the type of feeding

**DOI:** 10.1002/fsn3.938

**Published:** 2019-01-28

**Authors:** Edgar M. Vásquez‐Garibay, Alfredo Larrosa‐Haro, Elizabeth Guzmán‐Mercado, Nelly Muñoz‐Esparza, Samuel García‐Arellano, Francisco Muñoz‐Valle, Enrique Romero‐Velarde

**Affiliations:** ^1^ Instituto de Nutrición Humana Universidad de Guadalajara Guadalajara México; ^2^ Nuevo Hospital Civil de Guadalajara Dr. Juan I. Menchaca Guadalajara México; ^3^ Instituto de Investigación en Ciencias Biomédicas Universidad de Guadalajara Guadalajara México

**Keywords:** appetite regulatory hormones, breastfeeding, formula feeding, infants

## Abstract

Satiety and appetite‐stimulating hormones play a role in the regulation of food intake. Breastfed infants may have a different profile of serum appetite‐regulating hormones than formula‐fed infants. We propose to demonstrate that the serum concentration of appetite regulatory hormones differs according to the type of feeding and that there is a correlation between the serum concentrations of these hormones in mothers and in infants at 4 months of age. In a cross‐sectional analysis, 167 mother–newborn dyads at the Hospital Civil de Guadalajara were enrolled: 74 full breastfeeding (FBF), 56 partial breastfeeding (PBF), and 37 receiving human milk substitutes (HMS). Serum levels of ghrelin (pg/ml), leptin (ng/ml), peptide YY (pg/ml), and glucagon‐like peptide‐1 (GLP‐1) (pM) were measured. We performed one‐way analysis of variance, unpaired Student *t* test, post hoc Tukey test, and Pearson correlation. The total sample at 16 weeks postpartum included 167 dyads. The mean age was 16 ± 1 weeks. The concentrations of GLP‐1 (pM) and peptide YY (pg/ml) were higher in the FBF group (42.6 and 442.9) than in the HMS group (35.2 and 401.9), respectively, *p *=* *0.046 and *p *=* *0.056. And, the FBF group had higher correlation coefficients of ghrelin (*r *=* *0.411 vs. 0.165), GLP‐1 (*r* = 0.576 vs. 0.407), and peptide YY (*r* = 0.218 vs. 0.067), respectively, than the HMS group. The concentrations of GLP‐1 and peptide YY were higher in the FBF group when compared with the HMS group. Mother–infant dyads fed by FBF had more significant direct correlations of appetite‐regulating hormones than those who received HMS.

## 1 INTRODUCTION

The World Health Organization (WHO, 2016) recommends offering “exclusive breastfeeding for the first 6 months, the time when safe and nutritious foods are introduced while breastfeeding continues and can be extended to the second year of life.” The creation of the WHO growth standard of healthy breastfed infants supports the perception that growth and cognitive development are optimal when this standard is adhered to and that formula‐fed infants deviate from this reference (De Onis, Garza, Onyango, & Borghi, 2007; Victora et al., 2015). Hypothetically, exclusive breastfeeding for 6 months propitiates the ability to self‐regulate energy intake by the infants according to their needs, unlike formula‐fed infants (Brown & Lee, 2012; Park, Kim, Chu, Jekal, & Lee, 2015). It has also been noted that the protective role of breastfeeding against the development of obesity could be partially explained by the composition of human milk and also by the presence of appetite‐regulating hormones in breastfed infants (Li, Magadia, Fein, & Grummer‐Strawn, 2012; Petridou et al., 2005; Savino et al., 2008).

Appetite and body weight regulation are controlled by the central nervous system (CNS) in a rather complicated manner. In particular, the center of hunger in the hypothalamus is a nuclear region of the brain currently considered responsible for controlling appetite (Cassidy & Tong, 2017). However, most of the available evidence derives from experiments in rodents, and the role of this system in regulating appetite in states of hunger/starvation and in the pathogenesis of overeating/obesity remains to be fully elucidated in humans (Farr, Li, & Mantzoros, 2016).

It is accepted that satiety and appetite‐stimulating hormones play a role in the regulation of food intake and body composition by signaling satiety and energy reserves through hypothalamic receptors, during and after the lactation stage (Marić et al., 2014; Münzberg & Morrison, 2015). Appetite‐stimulating hormones, such as ghrelin, are important in the initiation, cessation, and frequency of eating (Breij, Mulder, van Vark‐van der Zee, & Hokken‐Koelega, 2017). Satiety‐regulating hormones, such as leptin, glucagon‐like peptide‐1 (GLP‐1), and peptide YY, decrease food intake, promote satiety, decrease the desire to eat, and increase the metabolic rate (Schueler, Alexander, Hart, Austin, & Larson‐Meyer, 2013). Breij et al. (2017) have hypothesized that formula‐fed infants could have a different profile of serum appetite‐regulating hormones than breastfed infants.

We might also hypothesize that partially breastfed infants would have a different profile of these hormones. Therefore, the purpose of the study was to demonstrate that the serum concentrations of leptin, ghrelin, GLP‐1, and peptide YY in mother–infant dyads differ according to the type of feeding. In addition, we were interested in exploring the correlation between the appetite regulatory hormones in mothers and their infants at the fourth month of postnatal life.

## 2 METHODS

### 2.1 Design

This is a cross‐sectional analysis of a sample of 167 mother–infant dyads according to type of feeding: 74 full breastfeeding (FBF), 56 partial breastfeeding (PBF), and 37 human milk substitutes (HMS). This sample was obtained from a nonrandom cohort study.

### 2.2 Sampling method

The sampling system was nonprobabilistic at the site of birth concentration. We identified 815 mother–newborn dyads who were admitted to the physiological puerperium ward in a shared room at the Nuevo Hospital Civil de Guadalajara. They were included and followed for 4 months if they met the inclusion criteria: postpartum healthy women living in the metropolitan area of Guadalajara who had signed the informed consent sheet, with a full‐term healthy single infant of either sex who had an adequate weight for gestational age. Dyads were not included when mothers had a history of chronic, genetic, or congenital diseases; addiction to alcohol, tobacco, or drugs; or if newborns had congenital malformations and/or genetic diseases. Dyads were also excluded for maternal causes such as loss of follow‐up, presence of subacute or chronic disease, occurrence of illness or serious accident, and/or infant causes such as subacute or chronic disease, occurrence of an accident or serious illness, or incomplete data of the mother or infant. A total of 408 dyads were contacted by telephone at the fourth week of postnatal life; 219 dyads accepted and attended the first visit at the eighth week, and 170 dyads attended at the end of the 16th week of postnatal life. Three dyads were excluded for incomplete information; thus, the final sample included 167 dyads. There were no significant differences in the general characteristics of the mother–infant dyads between study participants and mothers who were not located by telephone at 4 weeks or those who declined to participate in the study at 8 weeks postpartum. With averages and variances of serum leptin from nursing and nonlactating mothers (Larson‐Meyer, Ravussin, Heilbronn, & DeJonge, 2010), an alpha of 0.05 and a power of 0.80, a total sample size of 148 dyads was estimated.

### 2.3 Dependent variables

Dependent variables included serum appetite‐regulating hormones: total ghrelin (pg/ml), leptin (ng/ml), peptide YY (pg/ml), and GLP‐1 (pM).

### 2.4 Independent variables

The study's independent variables included FBF, PBF, and HMS based on cow's milk.

### 2.5 Other variables

Other variables were weight (g) and height (cm).

### 2.6 Collection of blood and assays

At 16 weeks of postnatal age, blood samples were obtained from the mother (3 ml) and the infant (0.5 ml) during fasting (12 hr for mothers and 4 hr for infants). To stabilize appetite‐regulating hormones, blood samples were placed in Vacutainer® EDTA anticoagulant tubes treated with aprotinin protease inhibitor (500 KIU/ml, EMD Millipore, Billerica, MA, USA) and inhibitor of dipeptidyl peptidase‐4 (10 μl/ml, EMD Millipore). After collection, plasma was separated by centrifugation at 3000 *g* for 15 min at 4°C, aliquoted in 0.6‐ml tubes, and then stored at −80°C. The leptin hormones (R&D Systems, Minneapolis, MN, USA), ghrelin, peptide YY, and GLP‐1 (all three from EMD Millipore) were analyzed with commercial enzyme‐linked immunosorbent assay kits. For the determination of leptin, the samples were diluted 1:20. The sensitivity of the assay for leptin, ghrelin, peptide YY, and GLP‐1 was 7.8 pg/ml, 30 pg/ml, 6.5 pg/ml, and 1.5 pM, respectively. The concentrations of the hormones were determined from curves generated from the standards of each kit, using a logistic regression model of four parameters.

### 2.7 Measuring instruments and techniques

#### 2.7.1 Weight

Infants were placed naked and without a diaper on a weighing scale (model 314, Seca, Hamburg, Germany). Weight was measured with a precision of 10 g.

#### 2.7.2 Length

The measurement of length was obtained by two observers using an infant bed (model 416, Seca). The measurement had an accuracy of 0.1 cm.

### 2.8 Field work criteria and strategies

The study began in the Physiological Puerperium of the Gyneco‐obstetrics Division of the Hospital Civil de Guadalajara. Mothers were invited to participate after researchers (EGM, NME) promoted FBF for at least 6 months. We clarified that we were interested in including all the mothers who wanted to participate regardless of the mode of feeding that they chose for their infants. Those who agreed to participate were scheduled appointments at 16 weeks postpartum. A fasting blood sample was taken from the mother–infants dyads.

### 2.9 Collection of information, databases, and computer programs

Once the information was obtained, the database was elaborated, the data were captured, and the statistical analysis was performed with SPSS version 24 software.

### 2.10 Statistical analysis

We used Levene's test to assess equality of variances, and for two or more groups, and Shapiro–Wilk and Kolmogorov–Smirnov tests for exploring the normality of distributions. We performed analysis of variance for comparison of variances among groups, post hoc Tukey test for repeated measures, and unpaired Student *t* tests to show the contrast between two independent samples with normal distribution. In variables with non‐normal distribution, the Mann–Whitney *U* test on samples was used. Linear regression and Pearson correlation coefficient between parametric variables and Spearman correlation between nonparametric variables with very wide variances were also obtained. The level of significance was a *p *≤* *0.05.

### 2.11 Biosecurity

The handling of the biological samples was carried out according to the specifications mentioned in the Mexican Official Standard NOM‐087‐ECOL‐SSA1‐2002. The chemical substances were handled and stored in accordance with Mexican Official Standards NOM‐052‐SEMARNAT‐2005 and NOM‐054‐SEMARNAT‐1993, in addition to information indicated in the biosafety sheets for each chemical substance used in each experiment.

## 3 RESULTS

The total sample at 16 weeks postpartum included 167 dyads. The age of the infants in the three groups based on type of feeding was 16 ± 1 weeks. The mean age of the mothers was 23.7 ± 4.6, 23.5 ± 4.6, and 22.7 ± 4.0 years, and the mean age of the fathers was 27.1 ± 6.2, 27.1 ± 5.4, and 27.8 ± 6.6 years in the FBF, PBF, and HMS groups, respectively. The infant weight was 6642 ± 722, 6,333 ± 589, and 6,179 ± 855 g, and infant length was 62.2 ± 2.0, 62.1 ± 1.6, and 61.7 ± 2.2 cm, respectively, with no significant differences among groups.

There were no differences in the concentration of total ghrelin and leptin in the 4‐month‐old infants among the three groups. The post hoc analysis showed that the concentration of GLP‐1 was significantly higher in the FBF group than in the HMS group (*p *=* *0.046). The concentration of peptide YY also showed a higher concentration in the FBF group than in the HMS group (*p *=* *0.056), Table 1. In the group of mothers of 4‐month‐old infants according to the type of feeding, only the leptin concentration showed significant differences among the three types of feeding. The bivariate comparison (Student's *t* test) showed that the concentration was higher in the group of FBF vs PBF (*p *=* *0.012) and in the group FBF vs HMS (*p *=* *0.029), Table 2.

**Table 1:** Comparison of the serum concentration of appetite‐regulating hormones measured in 150 four‐month‐old infants according to the type of feeding^a^

Appetite‐regulating hormones	FBF (*n *=* *66)		PBF (*n *=* *50)		HMS (*n *=* *34)		*p*
	*x*	*SD*	*x*	*SD*	*x*	*SD*	
Total ghrelin (pg/ml)	776.6	373.4	849.9	359.7	907.9	336.6	0.208
Leptin (ng/ml)	3.1	1.6	3.0	1.5	2.8	1.9	0.829
GLP‐1 (pM)	42.6	19.6	44.1	19.9	35.2	15.2	0.091
Peptide YY (pg/ml)	442.9	103.9	419.2	89.1	401.9	98.9	0.120

Notes

Bivariate comparisons (Student's *t* test); FBF: GLP‐1 42.6 ± 19.6 versus HMS (*n *=* *34) 35.2 ± 15.2, *p *=* *0.046; Peptide YY: FBF 442.9 ± 103.9 versus HMS 401.9 ± 98.9, *p *=* *0.056. Some outlier's values were eliminated.

FBF: Full breastfeeding; GLP‐1: Glucagon‐like peptide‐1; HMS: Human milk substitutes; PBF: Partial breastfeeding.

a One‐way ANOVA.

**Table 2:** Comparison of the serum concentration of appetite‐regulating hormones measured in 161 mothers of 4‐month‐old infants according to the type of feeding^a^

Appetite‐regulating hormones	FBF (*n *=* *70)		PBF (*n *=* *55)		HMS (*n *=* *36)		*p* a
	*x*	*SD*	*x*	*SD*	*x*	*SD*	
Total ghrelin (pg/ml)	362.2	170.9	339.4	159.3	363.9	144.7	0.683
Leptin (ng/ml)	11.0	7.6	15.2	8.2	14.5	8.4	0.009
GLP‐1 (pM)	33.1	16.5	35.8	15.7	33.9	16.8	0.645
Peptide YY (pg/ml)	194.5	53.8	208.9	51.1	186.5	50.3	0.113

Notes

Post hoc test (Tukey): Leptin, FBF versus PBF, *p *=* *0.012; FBF versus HMS, *p *=* *0.077. Bivariate comparisons (Student's t test); Leptin, FBF vs, PBF, *p *=* *0.004; FBF versus HMS, *p *=* *0.029. Some outlier's values were eliminated.

FBF: Full breastfeeding; GLP‐1: Glucagon‐like peptide‐1; HMS: Human milk substitutes; PBF: Partial breastfeeding.

a One‐way ANOVA.

Table 3 shows the linear bivariate correlations of the serum concentration of appetite‐regulating hormones in 4‐month‐old mother–child dyads according to the type of feeding. There was a significant high correlation of ghrelin (*r *=* *0.411, *p *<* *0.001) and GLP‐1 (*r *=* *0.576), *p *<* *0.001) and a nonsignificant trend correlation with peptide YY (*r *=* *0.218, *p *=* *0.081) in the FBF group; a nonsignificant trend correlation of ghrelin (*r *=* *0.254, *p *=* *0.064); a significant high correlation of GLP‐1 in the PBF group (0.378, *p *=* *0.006); and a significant high correlation of GLP‐1 (*r *=* *0.407, *p *=* *0.029), but no correlation with ghrelin, leptin, and peptide YY in the HMS group.

**Table 3:** Linear bivariate correlations of the serum concentration of appetite‐regulating hormones in 4‐month‐old mother–child dyads according to the type of feeding

Type of feeding	Appetite regulatory hormones	*r*	*p*
FBF (*n *=* *66)	Ghrelin	0.411	0.001
	Glucagon‐like peptide‐1	0.576	<0.001
	Peptide YY	0.218	0.081
PBF (*n *=* *50)	Ghrelin	0.254	0.079
	Glucagon‐like peptide‐1	0.378	0.006
	Peptide YY	0.099	0.488
HMS (*n *=* *34)	Ghrelin	0.165	0.357
	Glucagon‐like peptide‐1	0.407	0.029
	Peptide YY	0.067	0.711

Notes

Leptin did not correlate significantly in any of the cases. Some outlier's values were eliminated.

FBF: Full breastfeeding; HMS: Human milk substitutes; PBF: Partial breastfeeding.

## 4 DISCUSSION

In relation to appetite‐regulating hormone biomarkers, similarities and important differences were observed between infants receiving FBF and those receiving HMS. There were no differences in the serum concentration of total ghrelin and leptin among the three infant groups. However, the serum concentration of GLP‐1 was significantly higher in the FBF group than in the HMS group (*p *=* *0.046); the serum concentration of peptide YY was also higher in the FBF group than in the HMS group. Our results regarding the serum concentrations of leptin and peptide YY in infants with the three types of feeding were higher than those reported by Breij et al. (2017). We do not have a clear explanation for these differences, since the participants in the Breij et al., study were 3 months old and our participants were 4 months old. The differences in ghrelin were obvious because we measured total ghrelin and they measured activate ghrelin.

Some of the observed results could be related to the physiological functions and mechanisms of action of these two appetite‐regulating hormones both in the gastrointestinal tract and in the CNS. It is known that the peripheral melanocortin 4 receptor, which has an essential role in energy regulation, is implicated in the regulation of GLP‐1 and peptide YY (Breij et al., 2017; Choudhury, Tan, & Bloom, 2016). The peptide hormone GLP‐1 is derived from the transcription of a gene called pro‐glucagon, whose physiological function is based on reducing blood glucose concentration through increased secretion of insulin and suppression of glucagon secretion by the pancreas (Meier et al., 2004; Schueler et al., 2013). Among its other functions, GLP‐1 inhibits gastric acid secretion and gastric emptying and also suppresses food intake through the sensation of satiety. In the CNS, it increases the acquisition and strength of conditioned aversions to taste, anxiety, nausea, or visceral discomfort. In the presence of GLP‐1, the pleasurable value of food, the motivation (reward) for eating, and the amount and frequency of food consumption decrease (Graaf et al., 2016; Skibicka, 2013). Peptide YY inhibits gastric motility; consequently, it increases the efficiency of digestion and nutrient absorption after a meal and also increases the absorption of water and electrolytes in the colon. Breij et al. (2017) have reported that breastfed infants have higher peptide YY concentrations and have speculated that it could be a link to the protective role against obesity in exclusive breastfeeding.

Among the mothers of these 4‐month‐old infants, only the concentration of leptin showed significant differences among the three types of feeding. The bivariate comparison showed that the leptin concentration was lower in the FBF group as compared with the PBF group, as well as in the FBF group compared with the HMS group. It is probable that the high serum leptin concentration in mothers of the HMS group was more a function of excess of adiposity. However, the concentration of total ghrelin in mothers of infants in the three types of feeding groups was lower than that reported by Gibbons et al. (2013) in adults of both sexes who were overweight or obese. Likewise, the concentration of GLP‐1 and the concentration of peptide YY are higher in our groups. These differences mean that the mothers in this immediate postnatal stage conserve metabolism and hormonal appetite regulation (orexigenic and anorexigenic), which differs from adult individuals with overweight or obesity.

The linear bivariate correlations of the serum concentration of appetite‐regulating hormones in 4‐month‐old mother–child dyads according to the type of feeding showed a highly significant direct correlation of ghrelin and GLP‐1 and a mild correlation with peptide YY in the FBF group. A mild correlation of ghrelin and a high correlation of GLP‐1 was observed in the PBF group, and a high correlation of GLP‐1 was seen in the HMS group. The marked differences between the correlations of the mother–child dyads in the FBF group vs the HMS group show the potential influence that breastfeeding has on the appetite‐regulating hormones of their infants (Ghrelin, GLP‐1, and peptide YY), as has been amply pointed out by Savino, Benetti, Liguori, Sorrenti, and Cordero Di Montezemolo (2013).

The main limitation of the study was that the number of infants fed HMS was lower than those who received FBF and PBF. The main strength was the demonstration of differences in the concentration of appetite‐regulating hormones, particularly between the group who received FBF and the group who received HMS.

In conclusion, we found higher serum concentrations of GLP‐1 and peptide YY in FBF infants of 4 months of age than those who received HMS. The serum concentration of leptin was significantly higher in mothers of the HMS group versus the FBF group, probably because of higher adiposity in the HMS group. In addition, mother–infant dyads fed by FBF had more significant direct correlations of appetite‐regulating hormones than those who received HMS, probably influenced by human milk.

## CONFLICT OF INTEREST

None.

## ETHICAL CONSIDERATIONS

The recommendations of the Declaration of Helsinki were followed in its last amendment during the 64th Annual Assembly organized by the World Medical Association (2013). The protocol was approved by the Committees of Bioethics and Research of the Hospital Civil de Guadalajara and the Committees of Biosecurity, Bioethical and Research of the University of Guadalajara, Center of Health Sciences (CI‐01314). After the mother had given her authorization by signing the informed consent, the protocol was applied to each of the participating dyads who met the inclusion criteria.
